# Characterization of natural HIV-1 Tat and Vpr variants from Northern India

**DOI:** 10.1186/1471-2334-12-S1-P8

**Published:** 2012-05-04

**Authors:** Sneh Lata, Vikas Sood, Sajad A Dar, Akhil C Banerjea, Shukla Das, VG Ramachandran

**Affiliations:** 1 University College of Medical Sciences and Guru Teg Bahadur Hospital, Delhi, India; 2 National Institute of Immunology, New Delhi, India

## Background

HIV-1 Tat & Vpr are multifunctional and involved in transactivation, cell cycle regulation, MHC-1 modulation etc. Like other HIV genes, Tat and Vpr are subject to variation. Recombination frequency is higher in the first exon of Tat and Vpr. Characterization of these variants is the subject of the present study.

## Methods

HIV-1 Tat exon 1 and Vpr were amplified from the DNA isolated from blood of HIV infected patients and cloned. Clones were got sequenced and aligned against reference sequences using CLUSTAL W. Sim plot analysis was done for recombinants. Their expression was accessed by transfection of HEK 293T cells with myc fusion clones of variants and western blotting using anti-myc antibody. The variant Tat clones were co-transfected with LTR-luc to investigate their LTR transactivation potential by dual luciferase reporter assay.

## Results

Exon 1 of Tat was amplified from 21 samples and Vpr from 16 samples. Four Tat exon 1 and two Vpr sequences were found to have unique variations. Among the four unique Tat variants, one resembled subtypes B and C. This recombination in Tat was found to negatively affect its transacivation potential of reference strain B LTR in comparison with native Tat. Two Vpr sequences resembled subtypes B, C, and D at different locations. One Vpr variant had a frameshift towards C-terminus.

## Conclusion

Variations in Tat affect the functional aspects of the protein including interactions with other viral proteins with consequences for virus-host interaction.

